# The role of neutrophils in chorioamnionitis

**DOI:** 10.3389/fimmu.2023.1198831

**Published:** 2023-07-05

**Authors:** Cunling Zhang, Jiasong Cao, Meiyi Xu, Dan Wu, Wen Li, Ying Chang

**Affiliations:** Tianjin Key Laboratory of Human Development and Reproductive Regulation, Tianjin central hospital of Gynecology Obstetrics, Tianjin, China

**Keywords:** chorioamnionitis, neutrophils, maternal-fetal interface, preterm birth, preterm premature rupture of fetal membranes, fetal inflammatory response syndrome

## Abstract

Chorioamnionitis, commonly referred to as intrauterine infection or inflammation, is pathologically defined by neutrophil infiltration and inflammation at the maternal-fetal interface. Chorioamnionitis is the common complication during late pregnancy, which lead to a series of serious consequences, such as preterm labor, preterm premature rupture of the fetal membranes, and fetal inflammatory response syndrome. During infection, a large number of neutrophils migrate to the chorio-decidua in response to chemokines. Although neutrophils, a crucial part of innate immune cells, have strong anti-inflammatory properties, over-activating them can harm the body while also eliminating pathogens. This review concentrated on the latest studies on chorioamnionitis-related consequences as well as the function and malfunction of neutrophils. The release of neutrophil extracellular traps, production of reactive oxygen species, and degranulation from neutrophils during intrauterine infection, as well as their pathological roles in complications related to chorioamnionitis, were discussed in detail, offering fresh perspectives on the treatment of chorioamnionitis.

## Introduction

The key successful pregnancy is based on the exquisite modulation of the maternal immune system to allogeneic fetal tolerance (1). Depending on the stage of gestation, pregnancy alternates between pro- and anti-inflammatory process (2). During the first trimester of pregnancy, mildly pro-inflammatory milieu and the development of immunological tolerance are crucial for embryo implantation. The high concentration of cytokines (IL-6, IL-8/CXCL8, TNF-α) generated by endometrial cells and immune cells in the site of implantation in the first trimester attracts natural killer (NK) cells (65-70%), macrophages (10-20%), and dendritic cells (DC) (2-4%) to decidua (3, 4). To promote trophoblast invasion, NK cells generate the chemokines IL-8/CXCL8 and interferon-inducible protein-10. Additionally, decidual NK cells contribute significantly to the development of blood vessels by secreting angiogenic factors (5, 6). Early in pregnancy, T cells make up 10–20% of the decidual leukocytes (7) and the number of helper T(Th)1 cells and regulatory T cells (Tregs) also increase (8). Cytokines produced by Th1 cells, such as IL-6, IL-1, IL-15, IFN‐γ, and IL-8/CXCL8, have a role in minor inflammation, embryo implantation, trophoblast invasion, and the recruitment of immune cells (9–11). Human placental explants secrete G-CSF, IL-10, and TGF-β, which cause circulating monocytes and T cells to convert into M2 macrophage and Tregs in order to build maternal tolerance (12). Human leukocyte antigen (HLA) G, which is expressed on the surface of extravillous trophoblast, interacts with receptors on maternal immune cells like T cells, NK cells, and DC cells to modify maternal-fetal immunological tolerance (13). An amount of Myeloid-derived suppressor cells (MDSCs) are seen in the maternal-fetal interface during healthy pregnancy. In order to exert an immunosuppressive impact and participate in maternal-fetal immunological tolerance, studies have shown that MDSCs interact with NK cells, DC, and Tregs through enzymes and cytokines (14). Recent study discovered innate lymphoid cells (ILCs) in the uterus have the capacity to sustain maternal immunological tolerance (15).

Neutrophils, the most common type of white blood cell, are crucial in a number of pregnancy issues. Periodontitis is a dysbiotic inflammatory disease that is caused by gram-negative microaerophilic and anaerobic bacteria, which has been linked to preterm birth, fetal growth restriction, preeclampsia, and gestational diabetes mellitus (GDM) (16–18). During periodontitis, numerous neutrophils are consistently drawn to the subgingival crevice (19). Studies have shown that the number of neutrophils is positively correlated with the severity of periodontitis (20, 21). In preeclampsia patients, neutrophils are activated with oxidized lipids generated by the placenta which inducing ROS, TNF-α, and myeloperoxidase (MPO), harming the vascular endothelium (22–24). Neutrophils that are persistently recruited and defective induce tissue injury and continuous inflammation (19, 25). In GDM cases, a significant quantity of neutrophil infiltration was found in placentae and overt neutrophils produced increased NETs and NE, pointing to the association between neutrophils and GDM (26).

Intrauterine infection or inflammation can prematurely trigger the pro-inflammatory signaling, which is clearly linked to the disturbance of immune tolerance in maternal-fetal interface and a series of pregnancy-associated complications (27–29). Clinical signs of chorioamnionitis (both acute and chronic) include maternal fever, leukocytosis, uterine discomfort, and fetal tachycardia (30). The histological definition of chorioamnionitis is the infiltration of neutrophils in the chorion and/or amnion (31). In the third trimester, NK cells (20%), macrophage (20–30%), T cells (20–30%) and neutrophil (10–20%) make up the majority of the white cells in the maternal–fetal contact. During chorioamnionitis, more than 60% of the leukocytes present are neutrophils, while the percentages of macrophages, NK cells, and T cells are 10%, 10%, and 15%, respectively (32). Neutrophils are vital component of the innate immune system, performing a key role in eradicating microorganisms that translocate across the epithelium and infiltrate the mucosa. Neutrophils employ NETs, reactive oxygen species (ROS), and antimicrobial enzymes, such as defensins, neutrophil elastase (NE), and myeloperoxidase (MPO) to modify the inflammatory response and fight microorganisms (33).

Chorioamnionitis is a complex inflammatory process that can produce a variety of pro-inflammatory factors, leading to various complications. Over-recruitment and response of neutrophils during chorioamnionitis can potentially damage the FMs, leading to a number of problems. Neutrophil-derived inflammatory cytokines (TNF-α, IL-8/CXCL8, and CCL4/MIP-1β) are involved in preterm labor. Matrix metalloproteinases (MMPs) released from neutrophils can make contribution to premature rupture of fetal membranes (PROMs) through weakening collagen scaffolds. Moreover, intrauterine infections or inflammatory irritation may also lead to damage to the fetus, referring to the fetal inflammatory response syndrome (FIRS). The research is currently concentrated on the protective role of neutrophils in chorioamnionitis. However, excessive neutrophil activation might seriously harm the organism, which could be worse than the illness itself. This review aims to investigate neutrophil dysfunction in chorioamnionitis progression further. The function of neutrophils can be regulated by appropriate mechanisms to ensure their bactericidal clearance function without malicious effects on fetal membrane cells. It is possible to lessen or even completely prevent issues linked to chorioamnionitis by fine-tuning the neutrophil response.

## The causes and outcome of chorioamnionitis

The word “chorioamnionitis” denotes the possibility of an inflammatory or infectious condition during pregnancy affecting either the chorion or the amnion, or both (34). Inflammation at the chorionic and amniotic membranes, along with maternal symptoms, can be used to identify chorioamnionitis histologically and clinically (35). There are several hypothesized methods by which bacteria spread to produce chorioamnionitis. The upward transmission of microorganisms from the lower to upper reproductive tract is the most common route. Only rarely do bacteria enter by invasive procedures or hematogenous transmission (36). Microorganisms invade the supracervical decidua and subsequently colonize the chorionic amnion, leading to infection in amniotic cavity and even the fetus. Increased amounts of pro-inflammatory cytokines are generated at the location of the infection, attracting plenty of neutrophils to the placenta and FMs (36). A healthy amount of Lactobacillus can be found in the vagina (37). Dysbiosis develops as the amount of Lactobacillus declines, and the variety of vaginal bacteria significantly increases (38), which can result in a number of pregnancy issues. In a prospective cohort study, researchers discovered that microbial diversity was associated with the occurrence of clinical chorioamnionitis and that the presence of Lactobacillus spp. could shield pregnant women against the condition (39). In another study, chorioamnionitis was likewise linked to the variety of the vaginal flora. The severity of chorioamnionitis increases with the diversity of vaginal microorganisms (40). Previously, it was thought that the cervix and placenta were sterile, but as research advanced, it was shown that the upper vaginal tract contained normal flora (41, 42). Intriguingly, oral cavity bacterial species colonized on the placenta have been linked to chorioamnionitis (43–46). A recent investigation found that the oral and urogenital commensals that have been linked to chorioamnionitis were present in the placenta (47). The microbiota in the upper vaginal tract of term healthy pregnancies, histological chorioamnionitis (HCA), and clinical chorioamnionitis (CCA) patients were examined using 16S rRNA sequencing. Microorganisms in the intrauterine environment significantly decreased in the CCA group (48). Chorioamnionitis has a significant association with preterm premature rupture of fetal membrane (PPROM), preterm delivery, and FIRS resulting in increased morbidity and mortality of neonatal (49, 50). Chorioamnionitis also remarkably threatens the mother, including postpartum hemorrhage, lack of uterine contractions, the increased risk of cesarean delivery, and rare complications (e.g., infectious shock, adult respiratory distress syndrome, and coagulation disorders) (35).

## Brief introduction of neutrophils

The majority of white blood cells in peripheral blood are neutrophils, which have a lobulated nucleus, an abundance of granules, and secretory vesicles in the cytoplasm (51). Neutrophils are terminally differentiated cells of bone marrow origin. Although some researches suggested that neutrophils may live longer (52), the lifespan of neutrophils circulating in the bloodstream is12-18h (53). Healthy human bone marrow can generate 1-2 x 10^11^ neutrophils each day (51). Neutrophils are the first leukocytes to migrate to the site of inflammation and remove pathogenic microorganisms in several ways. Neutrophils are crucial for the removal of pathogens, and their lack in humans can result in severe immunodeficiency, according to clinical and experimental research (54).

The initial immune cells to protect against invasive harmful microorganisms are neutrophils and macrophages. Numerous membrane receptors expressed by neutrophils are capable of detecting microbial infections and inflammatory signals. A large number of inflammatory stimulus signals produced from infection sites, including LPS from bacteria, chemokines produced by infected cells, and fragments of complement activation (54) interactive with receptors. Following activation, circulating neutrophils begin to slowly roll, crawl, and cross the membrane at the junction to reach the infection site. Then, neutrophils control invading pathogenic microorganisms through phagocytosis, degranulation, respiratory bursts, and formation of NETs (55).

The primary feature of chorioamnionitis is neutrophil accumulation at the maternal-fetal contact. The source of neutrophil in the chorio-decidua is controversial, some researches found that neutrophils may be predominantly of maternal origin (56, 57). While bacteria invades the amniotic cavity, neutrophils from fetus may play the major role (58). Another study revealed that neutrophils in the amniotic fluid may have fetal and maternal origins (59). The origin of neutrophils in FMs and amniotic fluid requires further study. An increasing amount of research has shown that the maternal-fetal interface is home to a variety of neutrophil phenotypes. Early in gestation, low-density neutrophils, or polymorphonuclear myeloid-derived suppressor cells (PMN-MDSCs), are seen in human decidua and are crucial for healthy pregnancy. A decreased number of PMN-MDSCs was noted to be associated with unexplained recurrent miscarriages (60). Lipid accumulation is important for the maintenance of immunosuppressive function in PMN-MDSCs. An important factor in determining the phenotype of neutrophil and PMN-MDSCs could be intracellular lipids. Decidua neutrophils in the second trimester showed high levels of vascular endothelial growth factor-A (VEGF-A), arginase-1 (ARG-1), and CCL2/MCP-1, all of which could promote angiogenesis (61). The anti-inflammatory polarized and resting states of neutrophils are crucial for pregnancy. The ability of neutrophils to exhibit an anti-inflammatory phenotype was discovered to be promoted by estrogen and progesterone (62, 63). In the decidua, neutrophils with anti-inflammatory phenotype can enhance circulating vascular proliferation using pro-angiogenic vascular endothelial growth factor (64, 65). Recently, a research team used Mass Cytometry to detect neutrophils in FMs at different gestational weeks. They discovered that there were two major populations of neutrophils, one of which displayed a higher degree of CD16 expression (66). The CD16 gene is highly expressed in mature neutrophils in healthy human blood (67). In head and neck squamous cell carcinoma patients, CD16^high^CD62L^dim^ neutrophil subsets were detected which had anti-tumor function (68). The diversity of neutrophils and functional variations in FMs require more study.

## Neutrophils infiltrate into fetal membranes during chorioamnionitis

At the maternal-fetal interface, neutrophils are first identified in the first trimesters of pregnancy (69), and the number of neutrophils increases in second trimester. According to a study, uterine epithelial cells secreted GM-CSF that drew neutrophils to the maternal-fetal interface (70). During term delivery, with the pro-inflammatory response prepared for delivery, a small amount of chemoattractant IL-8/CXCL8 was released from trophoblast cells, and a modest amount of neutrophils was infused into the FMs (71). Additionally, it was discovered that IL-8/CXCL8 was linked to neutrophil infiltration in the myometrium (72).

**Figure 1:**
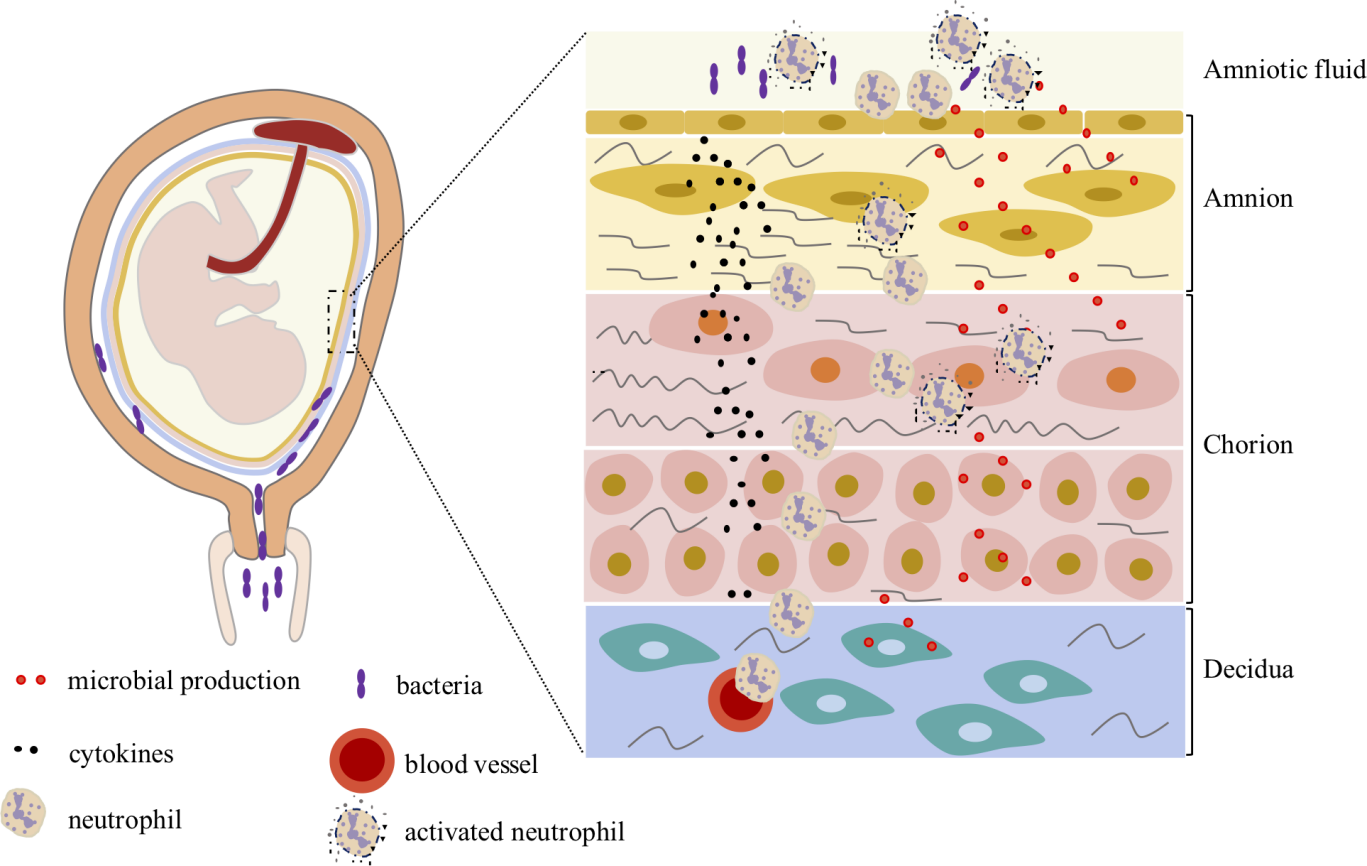
The neutrophils in fetal membranes during chorioamnionitis. When bacteria invade the maternal-fetal interface or even the amniotic fluid, neutrophils migrate from the decidua vasculature to the site of infection in response to the gradient of chemokines and pathogen-associated pattern molecules. Activated neutrophils clear pathogens in multiple ways.

Few neutrophils are seen during a healthy pregnancy, but chorioamnionitis causes a significant infiltration of neutrophils (**Figure 1**). The mechanism associated with massive neutrophil recruitment have not yet been fully clarified. Chemoattractants produced by the amnion may play a predominant role. Chemokines released from amniotic fluid establish a chemotactic gradient that attracts neutrophils to migrate to the chorion and amnion (73). Women experiencing infectious preterm labor had higher levels of IL-8/CXCL8 in their amniotic fluid (74). LPS was used in animal models to mimic infection in human pregnancy. For instance, in the rhesus macaque model, LPS was injected within the amniotic cavity. Similar to the phenomena seen in human chorioamnionitis, neutrophil-predominant immunological response was reported (75). The amnion secretes IL-1 during chorioamnionitis, which causes neutrophil accumulation via up-regulating the production of IL-8/CXCL8 and GCSF/CSF3. The recruitment of neutrophils in the chorio-decidua was decreased by using an IL-1 receptor blocker (32). Chemokines can also be secreted by trophoblast cells in addition to amniotic cells. For neutrophil migration to FMs, the higher expression levels of adhesion factors may be significant. In a macaque model with prolonged catheterization, the uterus was inoculated with GBS to mimic upstream infection. Rapid neutrophil accumulation in the chorionic membrane is accompanied by increased chemokine and neutrophil adhesion factor levels in FMs (such as L-selectin and ICAM-1) (76). FM immune cells interact with neutrophils in a cross-talk fashion. Neutrophils specifically respond to the cytokine IL-8/CXCL8, which was found in decidua during chorioamnionitis. Trophoblasts and macrophage-like cells were shown to generate IL-8/CXCL8 by immunohistochemistry analysis (77). During chorioamnionitis, pro-M2 convert was observed, and M2 macrophages have immunosuppressive qualities which means to minimization detrimental inflammation (78). Neutrophil activation can be modified by TNF‐α, IFN‐γ, IL-8/CXCL8, and GM-CSF generated by Th17 (79).

## The function of neutrophils in chorioamnionitis

Neutrophils, a crucial cell in the battle against pathogenic bacteria, have multiple strategies to get rid of pathogens. In response to bacterial stimulation, neutrophils produce large amounts of ROS during respiratory bursts. NETs are also a way to remove bacteria. Activated neutrophils can release antimicrobial substances by degranulation. Bioinformatics analysis of proteomics data was applied to the amniotic fluid during intra-amniotic cavity infection and histological chorioamnionitis. The amniotic fluid contains a variety of proteins, including histones H3, H4 (which are related to NETs), MPO (which are related to respiratory bursts), neutrophil gelatinase-associated lipocalin, and neutrophil defensin 1 (which is related to degranulation) (80).

## NETs formation in chorioamnionitis

NETs, consisting with decondensed chromatin and antimicrobial proteins (81), can be used to eliminate extracellular microorganisms. The bactericidal mechanism of NETs is mainly associated with the adhesion of pathogens to the reticulum and the killing of pathogens by higher local concentrations of antimicrobial peptides. Abundant NETs were found in FMs of women who suffered from chorioamnionitis (31). In the amniotic cavity, NETs are used by neutrophils to limit extracellular bacterial. Some scholars collected amniotic fluid samples from women with chorioamnionitis caused by bacteria infection to evaluate cell composition. They discovered an increase in neutrophils, the development of NETs, and the release of IL-1 (82). Studies found that bacteria activated neutrophils in FMs released NETs. GBS is associated with infection during pregnancy, which may cause chorioamnionitis. GBS vaginal infection in mice was applied to reveal the interaction of neutrophils and bacteria. To remove GBS, murine neutrophils induced NETs to restrict bacteria and antimicrobial molecules such as lactoferrin to inhibit bacterial growth (83). Polymicrobial stimulation such as LPS and poly (I:C) in FMs is common during chorioamnionitis. Multiple pathogenic infections in FMs were reported to increase the quantities and kinetics of NETs produced from neutrophils (84). In addition to bacteria, chemokines secreted from FMs modulate the formation of NETs. In another study, TNF-α secreted from LPS-stimulated FMs activated p38 MAPK pathway in neutrophil which enhanced the release of NETs (85). The release of NETs in FMs is crucial for the clearance of pathogens during chorioamnionitis.

Although the release of NETs is an effective method to remove extracellular pathogens, aggregation of NETs may also be deleterious. NETs can exert pro-inflammatory effects to damage different organs by promoting cytokine secretion and NLRP3 inflammasome activation (86–88). According to research, NETs activated the ROS-dependent mitochondrial pathway by using ERK1/2 signaling, which induced the apoptosis of trophoblast (89). The processes of cell death and fibrosis may be induced by substances containing NETs, such as extracellular DNA and histone exposure (90). NETs formation was observed in methicillin-resistant Staphylococcus aureus-caused bloodstream infection. DNase therapy reduced NET-related tissue damage to some extent (91). Poor outcomes were linked to the cell-free DNA from NETs that increased inflammation in septic patients by causing TNF-α release (92, 93). High levels of histones activated the TLR4 signaling pathway, promoting cellular injury and inflammation, which could aggravate multiple organ failure and fatal outcomes (94, 95). In chorioamnionitis, the formation of large amounts of NETs was observed, exerting the protective effect on the organism. However, excessive NETs have been found to cause severe damage to the organism in other disease studies, leading us to hypothesize that NETs may be detrimental to the management and prognosis of chorioamnionitis. It is essential to modulate the production of NETs in the appropriate range.

## ROS production in chorioamnionitis

Neutrophils produce large amounts of ROS during respiratory bursts. Nicotinamide adenine dinucleotide phosphate (NADPH) oxidase (NOX) complexes are assembled on phagosomal and cellular membranes with the stimulation of bacterial components or phagocytosis activity (96, 97). Using NADPH electrons, the oxygen molecule was changed into superoxide. Rapidly decomposing superoxide produces hydroxyl radicals and hydrogen peroxide, both of which are harmful to bacteria (98). During chorioamnionitis, large amounts of neutrophils are involved in FMs, which are one of the most important cells that produce ROS. LPS exposure in FMs not only promotes neutrophil recruitment but also increases ROS production. *In vitro*, LPS-stimulated FMs released some factors that significantly increased the production of ROS compared with unstimulated FMs. TNF-α is a pro-inflammatory cytokine that may affect the function of neutrophils. According to a publication, intra-amniotic injection of TNF-α alone in rhesus macaques has been reported to induce neutrophil recruitment in FM and preterm delivery (99). Some scholars found that TNF-α was associated with ROS production. The inhibition of TNF-α efficiently reduced LPS-induced ROS production in chorio-decidua neutrophils (75). Undoubtedly, the placenta may need to experience mild degrees of oxidative stress during development in order to control trophoblast invasion, differentiation, and proliferation as well as to promote placental angiogenesis. However, an excessive amount of oxidative stress may cause a variety of complications.

Oxidative stress may lead to oxidative damage, including peroxidation of membrane lipids, integrin, cytoplasmic proteins, and DNA. Increased ROS levels harm DNA in cancer cells, activating p53 and resulting in apoptosis (100). The irreversible end of the cell cycle is known as cellular senescence. Using ROS, neutrophil contact with human primary fibroblasts can speed up telomere dysfunction and early replicative senescence. The telomere shortening rate in hepatocytes during liver aging may be accelerated by ROS produced by neutrophils, leading to premature senescence (101). Circulating neutrophils produce more ROS during pregnancy compared to non-pregnancy. However, the effect of ROS on FMs has hardly ever been investigated. The senescence of FMs may be accelerated by intrauterine infection and inflammation, which can cause oxidative stress, DNA damage, and telomere shortening. It was reported that ROS could promote the apoptosis of trophoblast cells (49). ROS-induced cellular senescence was the p38 MAPK pathway depended (102) (103). Senescence of embryonic amniotic cells induced by oxidative stress was observed to be accompanied with activation of the p38 MAPK pathway (104).

At the FMs, trophoblast cells are crucial for coordinating immunological homeostasis. Vasoactive intestinal peptide (VIP), which is produced by trophoblast cells, has immunosuppressive and anti-inflammatory properties. According to a study, VIP produced by trophoblast cells prevented neutrophils from producing NETs and ROS (105). Trophoblasts are fetal epithelial cells having anti-inflammatory properties. A highly sensitive single-cell assay was used to analyze the interaction between neutrophils and trophoblasts. Trophoblasts used cellar contact to deactivate neutrophils, affecting glucose transport and metabolism while reducing ROS production in neutrophils and limiting oxidative DNA damage to nearby cells (106). ROS is a double-edged sword, meaning that the host benefits from its proper control.

## Granules release in chorioamnionitis

During phagocytosis, the fusion of phagosome in neutrophils promotes the release of antimicrobial substances and proteases stored in the granules which refers to degranulation. Four types of granules are observed in neutrophils (107–109), which are summarized as follows: 1) Primary granules consist of MPO, NE, proteinase 3, histone G, and defensins. These primary granules release antimicrobial proteins and proteases to kill pathogens. 2) Secondary granules are highly concentrated in antimicrobial compounds such antimicrobial peptide, lactoferrin, and lysozyme. 3) Gelatinase proteins make up tertiary granules. 4) Serum albumin and cytokines are found in secretory granules (109). The gradual release causes pathogenic damage, while limiting the exposure of host cells to cytotoxic molecules to reduce damage. The degranulation of neutrophils in FMs is a major method to protect the host. It was reported that degranulation was substantially promoted by LPS-stimulated FMs (85). CD63, a marker of primary granules release was enhanced by LPS. TNF-α inhibitors could reverse the increased level of CD63 on neutrophils, suggesting that TNF-α could regulate degranulation of neutrophils during chorioamnionitis (75). NE, a multifunctional serine protease stored in primary granules of neutrophils, has the ability to degrade proteins during phagocytosis. The NE concentration in the amniotic fluid significantly increased during amniotic cavity infection (110). Neutrophil MPO, which produces hypochlorous acid to kill pathogens during infection, is the most hazardous enzyme in neutrophils (111). Human FMs treated to modest concentrations of LPS increased neutrophil MPO degranulation *in vitro* (84). TNF-α has been implicated in degranulation as TNF-α antibody could reduce the expression levels of P-p38MAPK and P-ERK, influencing degranulation of MPO (85). Antimicrobial substances in granules are vital for neutrophils to fight against microbes, however, degranulation may also be fatal to the body.

Human neutrophils can either negatively or positively regulate cytokines using serine proteases (112). Combining chemokines with neutrophil granule proteins may improve binding affinity to receptors. Neutrophil-derived gelatinase B (MMP-9) may transform IL-8/CXCL8 into a more potent biological structure, promoting MMP-9 release. The inflammatory response may be amplified by this process (113–115). Notably, NE is the granule protein that may breakdown the extracellular matrix proteins which trigger chronic inflammatory disorders such rheumatoid arthritis and pulmonary emphysema (116, 117). Although few studies have concentrated on damage caused by neutrophil degranulation in FMs, the mechanism of chorioamnionitis warrants additional investigation.

## Dysfunction of neutrophils in chorioamnionitis-mediated complications

Preterm birth, which affects 5–18% of pregnancies worldwide, is defined as the delivery of the fetus before 37 weeks of gestation (118). It has been reported that preterm birth is the major reason for perinatal mortality and morbidity (119). More importantly, women who have preterm births run a high chance of having more preterm babies (120). Approximately 30% of preterm births are clinically diagnosed, indicating abnormal fetal or maternal conditions. The others are classified as spontaneous preterm births. Chorioamnionitis caused by intrauterine infection may explain approximately 40% of spontaneous preterm births (36). The process of preterm and full-term delivery has some similarities including sustained uterine contractions, cervical dilatation, and rupture of membranes during delivery (121). The shift of the uterus is associated with inflammatory mediators, such as cytokines (e.g., IL-8/CXCL8, IL-1, and IL-6), and contraction-associated proteins. Cervical dilatation is mediated by the increased expression levels of extracellular matrix proteins. Fetal membrane rupture is associated with the increased levels of cytokines, chemokines, and MMPs (29, 121).

Substantial evidence supports delivery as a pro-inflammatory process. However, it is essential to further clarify how the signaling leads to this inflammatory cascade. Histological studies have demonstrated that macrophages and neutrophils infiltrated into FMs during labor but the exact role of these cells has not been fully investigated. Increased levels of cytokines IL-1β, IL-6, IL-8/CXCL8, prostaglandins 2 (PTGS2), and TNF-α, which may be implicated in labor signaling, are linked to immune cell accumulation (122, 123).

Neutrophils are vital cells at the maternal–fetal interface, even though the precise role of neutrophils still need more investigate. In first trimester of pregnancy, neutrophils produce MMP9, ROS and hepatocyte growth factor (HGF) all of which involve in placentation and aid embryo implantation (124, 125). In addition, VEGF‐A, ARG‐1 and CCL2/MCP-1 produced by neutrophils are key player in remodeling and placental vascularization (11, 61). During term birth, abundant neutrophils have been seen to migrate to the myometrium (72), take part in cervical ripening, a crucial stage of labor (126–128), and produce pro-inflammatory cytokines and MMPs to aid in delivery (129)

RNA sequencing was used to reveal the immune response in FMs of women who have preterm labor with chorioamnionitis. Gene Ontology analysis indicated that biological pathways, including neutrophil activation, phagosome, leukocyte degranulation, and positive regulation of cytokine production, were enriched (130). Human neutrophil peptides 1-3 (HNP1-3) are α-defensins stored in primary granules of neutrophils (131, 132). Increased HNP1-3 was detected in amniotic fluid during chorioamnionitis-mediated preterm birth (133). The more severe chorioamnionitis is histologically associated with the higher levels of HNP1-3 (134). Neutrophils infiltrated in FMs mainly express pro-inflammatory cytokines, such as TNF-α, CCL4/MIP-1β, and IL-8/CXCL8 which make contribution to term and preterm parturition (74, 135–137). LPS-stimulated fetal membrane explants can release some inflammatory mediators that induce neutrophils to release abundance of cytokines, such as IL-17, IFN-γ, G-CSF, CXCL1/GRO-α, IL-10, CCL2/MCP-1, CCL3/MIP-1α, CCL4/MIP-1β, and RANTES/CCL5. This process is a positive feedback loop that may promote the migration of more neutrophils to the maternal-fetal interface (85). Rhesus macaque models of intrauterine infection were made with live *E. coli*. Neutrophil infiltration in the amnion expressed higher levels of IL6 and PTGS2 in *E. coli*-infected animals (138). IL-1β is recognized as a key cytokine in human and other animal models which is involved in chorioamnionitis related preterm birth (32, 99, 139). The increased IL-1β level is correlated with preterm birth and delivery in humans (31, 140). Neutrophil depletion reduced IL-1β level (141) suggesting that neutrophils may lead to preterm delivery in IL-1β-mediated way.

PPROMs occur in 30-40% of preterm birth cases (142, 143). PPROMs can be caused by various of factors, ultimately leading to the accelerated weakening of membranes. A large body of evidence, including results from clinical and basic studies, suggest that infection and inflammation are the major causes of PPROMs (144–146). Notably, PPROMs that occur at early gestational weeks are more likely to be caused by chorioamnionitis (147). Increased local cytokines, MMPs, collagenase, and protease activities may also cause PPROMs.

Neutrophil-derived MMPs can weaken the collagen scaffold, leading to PPROMs (148, 149). The primary source of tensile strength in FMs is collagen type I, which is degraded by MMPs or neutrophil collagenase (150). NETs in activated neutrophils contributed to the release of MMP-9 and prostaglandin E2 which mediated by TLR-9 recognized with DNA (151). Oxidative stress induced by various PPROMs-associated risk factors leads to premature aging of FMs, causing their dysfunction and structural weakening and rupture (80, 104, 152). It was reported that ROS could cause damage to collagens of the chorio-amnion in PPROMs. When subjected to ROS *in vitro*, FMs showed the same tissue modifications as PPROMs. Meanwhile, antioxidants inhibited the damage caused by ROS in chorio-amnion (153). Bioinformatics analysis of proteomics data in amniotic fluid revealed that oxidative stress-associated DNA damage and ROS generation were responsible for inflammation and proteolysis in PPROMs complicated with chorioamnionitis (80). Neutrophils use the sophisticated defense mechanism of degranulation to produce proteases and defensins to combat microorganisms. MPO levels in amniotic fluid were markedly increased when the amniotic cavity was infected. According to a study, MPO contributed to the pathophysiology of PPROMs (154). Furthermore, high concentrations of HNP1-3 in amniotic fluid were associated with PPROMs (133). Neutrophils are the main cells that can induce NETs, ROS, and degranulation during chorioamnionitis. As a result, the over-activation of neutrophils may contribute to PPROMs.

FIRS is the inflammatory response of fetus in answer to microbes or other stimuli which may lead to a series of complications in neonates (155). Vertical transmission indicates pathogen infected fetus derived from mother which is a major threat to the developing fetus. The fetus could suffer terrible effects from bacterial, viral, and parasite illnesses that are transmitted at the maternal-fetal interface (156). The placenta has strong defenses against infection in the case of vertical infection. However, microorganisms can cross the placental barrier in several ways, leading to fetal damage. In addition, bacteria infecting the amniotic fluid can rise through the perineum, vagina, cervix, abdomen, or fallopian tubes (157). A part of pathogen may be low virulent, such as normal flora, and others may be high virulent. During intra-amniotic infection, microbe invades fetus with fetal breathing, swallowing, skin, or ear which exert local or systemic inflammatory response. Fetal cytokine storm may result in various organ failure and even death once the systemic fetal inflammatory response is out of control. Chorioamnionitis can induce fetal inflammatory responses that are derived by neutrophils. In preterm infants born with FIRS, the levels of neutrophil-associated inflammatory proteins in the cord blood were elevated (158). Organs, such as the lung and brain, are affected by neutrophils during prenatal inflammation (159, 160).

The absence of infection was noted in certain FIRS cases, indicating that sterile inflammation in the amniotic cavity may possibly be a contributing factor to the disease (161–163). Chronic inflammatory conditions with a high level of CXCL10/IP10 expression, such as chronic chorioamnionitis, villitis of unknown etiology, and chronic deciduitis were found to be associated with FIRS (155). FIRS may develop in fetuses exposed to high cytokines. A crucial point is that cytokines can come from conditions other than infections, like tissue damage, cell death, etc. As for chorioamnionitis without microbial invasion of amniotic fluid, FIRS may be caused by the accumulation of neutrophils at FMs, cytokines released from neutrophils, and tissue damage from the excessive inflammatory response.

## The regulation of neutrophil function

Neutrophils, the most abundant innate cells in human blood, are one of most vital responders to the invading pathogen. Neutrophils use phagocytosis, ROS, NETs, and degranulation to destroy infectious threats. However, excessive infiltration and hyper-activated neutrophils can induce tissue damage in FMs during chorioamnionitis. Cytokines, proteases, ROS, and NETs released from neutrophils can also be culprits of tissue damage. It is essential to properly control the activation and function of neutrophils. ROS can be crucial for pathogen elimination and essential signaling molecules for neutrophil responses, including priming, degranulation, apoptosis, and the release of NETs. However, during chorioamnionitis, overwhelming infiltration and hyper-activated neutrophils might cause tissue injury in FMs. Excessive ROS production at FMs can cause cellular senescence and lead to PROMs or even preterm delivery. It is suggested to take appropriate measures to reduce or inhibit damage caused by ROS. In patients with COVID-19, the marker of oxidative stress could be used to identify the severity of the disease. Antioxidants are new avenues to target on excessive ROS production and N-acetyl-l-cysteine and vitamin C or combination with elastase inhibitors (e.g., sivelestat) are the candidates (164, 165). Excessive ROS produced by NOX_2_ via the pentose phosphate pathway during acute respiratory distress syndrome may exacerbate inflammation leading to host damage. Using the small molecules LDC7559 and NA-11, the pentose phosphate pathway can be inhibited to reduce NOX_2_-dependent ROS (166). As previously indicated, blocking TNF- α may also be a suitable tactic for inhibiting ROS production (75, 167). These drugs can be used to reduce the production of ROS to avoid host damage and also to ensure the clearance of pathogens.

The vital stage in the formation of NETs is the citrullination of histones, which Peptidyl Arginine Deiminase 4 (PAD4) is involved in (168, 169). Targeting PAD4 activity is a desirable approach to control NET formation. Several PAD4 inhibitors have been created and evaluated in preclinical and clinical investigations, showing promising results in reducing NETs formation and alleviating disease symptoms. BB-Cl-amidine, the inhibitor of PAD4 was used in mouse models to suppress NETs formation which relieved the injury in vascular and endothelial (170). The inhibition of PAD4 activity in murine neutrophils by GSK484 can suppress the formation of thrombosis caused by NETs (171). Inhibition of NETs formation may reduce inflammatory damage under endotoxic stress. During healthy pregnancy, NETs are barely detectable at the maternal-fetal interface. During chorioamnionitis, neutrophils produce large amounts of NETs, but there are rare studies on treatment with PAD4 inhibitors. The effect of PAD4 inhibitors on disease progression and prognosis of chorioamnionitis can be explored in a mouse model.

Degranulation can release the most toxic protein stored in primary granules which is depended on the interaction between Rab27a and synaptotagmin-like protein 1 (JFC1) (116, 172, 173). Rab27a belongs to the Rab family of small GTPase proteins localizing on azurophilic granules (174). JFC1, contains an amino-terminal Rab-binding domain which is used to bind Rab27a. JFC1 and Rab27a co-localize at the azurophilic granule membrane, which is dominant in neutrophils (175). Targeting the Rab27a-JFC1 interaction refers the promising direction to modulate degranulation (116). Nexinhibs, small-molecule inhibitors, disturb interaction of JFC1-Rab27a reducing azurophilic granule release without affecting neutrophils viability and other function such as phagocytosis, NETs production (173). Granule protein‐mediated tissue damage indicates that suppression of the function of granule proteins is another promising therapeutic approach. Recombinant α1‐proteinase inhibitor, the endogenous elastase inhibitors is available, and the part of inhibitors are evaluated in clinical trials (176, 177). Degranulation occurs following activation of neutrophils in chorioamnionitis, and the effects of degranulation on the mother and fetus have been little studied. The peculiarities of pregnancy lead to more caution in the use of drugs, and studies regulating neutrophil degranulation have not been seen. Based on studies in other diseases, we hypothesize that neutrophil degranulation may cause damage to the mother and fetus while protecting the organism. Chorioamnionitis caused by neutrophils may be rescued using inhibitors to control the degree of neutrophil activation.

The current study found that when chorioamnionitis occurs, neutrophils protect the organism by forming NETs, producing ROS, and degranulation. However, damage to the maternal-fetal interface by over-activated neutrophils has been little studied. Neutrophil activation products can cause damage to the organism, and these have been demonstrated in other tissues and organs, so the role of neutrophils in chorioamnionitis may be twofold, with appropriate activation being protective for the organism and excessive activation producing a poor prognosis which suggests a novel area of research. Targeting the different functions of neutrophils may also be one of the directions for the treatment of chorioamnionitis, but more studies are needed to provide evidence due to the specificity of medication during pregnancy.

## Conclusions

Chorioamnionitis, as a common obstetric disease, may induce a series of complication on mother and fetus. Clinical symptoms or histological findings are the main evidence to diagnose chorioamnionitis. However, the disease has already progressed to a more severe stage, by the time pregnant women present with clinical symptoms. After its diagnosis, the main treatment options include the use of broad-spectrum antibiotics, antipyretics, supportive therapy, and accelerated delivery. Antibiotics are one of the mainstays of current treatment, while they are highly ineffective in preventing the disease, partly because residual inflammation can lead to fetal and maternal damage. The role of neutrophils in the pathogenesis of chorioamnionitis is undeniable. Chorioamnionitis caused by neutrophils and the effects of neutrophil-secreted cytokines on preterm birth and PPROMs represent areas of active investigation. The findings may enable scholars to better understand the pathogenesis of chorioamnionitis and develop new therapeutics, thus promotion the treatment and prevention of the disease.

Excessive production of proteases, ROS, and inflammatory cytokines by neutrophils during pathogenic clearance may cause damage to FMs and fetus. It may cause preterm labor, accelerated aging of fetal membranes, rupture, fetal inflammation, etc. Various functions of neutrophils can be regulated to reduce the production of these substances and weaken damage to the host. To date, some drugs that target the function of neutrophils have been described in clinical trials, indicating one of the directions of treatment of chorioamnionitis. In addition, cells in FMs, such as trophoblast cells, can protect the maternal-fetal interface from damage by inhibiting neutrophil overreaction in several ways. However, not all pregnant women who develop chorioamnionitis have poor outcomes, suggesting that abnormal function of FMs may lead to the diminished regulation of neutrophils. The interaction between FMs and neutrophils is also a key component in the study of chorioamnionitis.

## Author contributions

All authors listed have made equal contribution to the work, and all authors have read the manuscript and approved it for publication.
